# Research on the Bearing-Deformation Characteristics of Prestressed Concrete Thin-Walled Pipe-Pile Cement-Soil Cushion-Composite Foundation

**DOI:** 10.3390/ma19214611

**Published:** 2026-10-30

**Authors:** Yaohui Yang, Fangyuan Gong, Gongfeng Xin, Yafan Yang, Qinghua Wang

**Affiliations:** 1 Shandong High-Speed Group Innovation Research Institute, Jinan 250014, China; yangyaohui1905@163.com (Y.Y.); gfxin@163.com (G.X.); 2 School of Civil and Transportation Engineering, Hebei University of Technology, 5340 Xiping Road, Beichen District, Tianjin 300401, China; 202431605031@stu.hebut.edu.cn; 3 Provincial Communications Planning and Design Institude Group Co., Ltd., Tianchen Road 2177, Jinan 250101, China; wqh_sdcpd@aliyun.com

**Keywords:** highway engineering, composite foundation, prestressed concrete thin-walled pipe pile, cement soil cushion, bearing deformation characteristic

## Abstract

Graded gravel or gravel is commonly used as the cushion material in composite foundations reinforced with prestressed concrete thin-walled pipe pile. For the road section without drainage requirements in highway engineering, it is proposed to replace gravel or the gravel cushion with cement-soil cushion, which can greatly reduce the project cost, but its engineering characteristics need to be further studied. Field test, numerical calculation and theoretical analysis were carried out on the composite foundation of prestressed concrete thin-walled pipe-pile cement-soil cushion. The results show that when the cement-soil cushion reaches a certain thickness, the settlement-control effect of composite foundation is better than that of gravel cushion. With the increase in cement-soil cushion thickness, cement content, pile length and pile-cap size, the pile-soil stress ratio increases, and the pile-soil settlement decreases. The larger the pile spacing is, the larger the pile-soil settlement is. Conversely, larger pile spacing leads to increased settlement. Among the influencing factors, the thickness of the cement-treated soil cushion is identified as the most significant parameter affecting the load-bearing and deformation behavior of the composite foundation. Beyond this apparent diminishing-return thickness, further increases in cushion thickness yield diminishing returns in settlement control. The settlement-control effect of double-layer reinforced-cushion-composite foundation is better than that of single-layer reinforced cushion. The position of reinforcement layer also has an important influence on the bearing deformation characteristics of composite foundation. Under the condition of single-layer reinforced cushion, placing the reinforcement at the bottom of the cushion proves more effective than at the top. From the perspective of settlement-control effect and economy, there is an optimal value for the ratio of pile spacing to pile-cap size, which is about 0.5. The above research results offer experimental data and practical engineering references for prestressed concrete thin-walled pipe-pile composite foundations with cement-soil cushions under the geological and loading conditions examined in this work.

## 1. Introduction

Improper treatment of soft soil foundation in highways will cause differential settlement or subsidence of road surface, cause bridgehead jumping and other phenomena, affect road driving comfort, and may also cause major road-safety accidents [1]. The commonly used foundation-treatment measures in road engineering include prestressed concrete pipe pile (PC pile), cement fly ash gravel pile (CFG pile), cement-soil mixing pile (DMC pile), etc. Among them, cement-soil mixing pile is widely used because of its low cost, convenient construction and small additional stress on the foundation [2]. However, there are also some problems in the technology of cement-soil mixing pile. Taking the deep cement mixing pile as an example, there is a common problem that the quality of pile forming in shallow foundation is good, and the quality of pile forming in deep foundation is poor [3,4,5,6]. The phenomenon that a large amount of cement slurry gushes out of the ground during the construction process shows that the cement slurry fails to fully mix with the foundation soil. Prestressed concrete pipe-pile composite foundation has many advantages, such as good reinforcement effect, reliable quality and fast construction speed [7]. However, its high engineering cost limits its large-scale application in engineering, to a certain extent [8].

According to the investigation and analysis, in the composite foundation of prestressed concrete pipe-pile networks, the cost of pipe pile accounts for about 50% of the total cost of composite foundation, pile cap accounts for 25%, and gravel or gravel cushion accounts for about 25%. Replacing the granular material with a cement-treated soil layer can substantially reduce the overall cost, thereby facilitating broader engineering application of such composite foundations [9,10,11]. With regard to bearing characteristics and numerical analysis of composite foundations, Priyanath et al. [12,13] conducted a comparative study of 2D and 3D finite-element numerical calculation models for prestressed concrete pipe-pile reinforced cushion-composite foundation under embankment loading. By comparing with the measured values, it is found that the calculation results of the 3D model are closer to the measured values, and have more reliable accuracy than the 2D model. It is proposed that the creep of the reinforcement layer reduces the vertical stress transferred to the pile top, while increasing the settlement of the intervening soil. FEI Kang [14] also used 2D and 3D finite-element calculation models to analyze the bearing characteristics of pile-supported embankments, and proposed that the 2D model can fully describe the parabolic and circular geometric-deformation shapes of geosynthetics. Whereas the three-dimensional model fails to reasonably reflect the actual three-dimensional behavior of the composite foundation. Matthieu et al. [15] investigated the bearing deformation characteristics of pile-supported reinforced embankment through a centrifuge test, with particular attention to the effects of gravel-cushion thickness and pile spacing. Their results indicate that the deformation deflection of gravel cushion decreases with increasing cushion thickness, and the differential settlement on the surface of gravel cushion is also reduced as the cushion thickness increases. Pile spacing exerts a more significant influence on the load-bearing capacity of the piles, whereas the effect of cushion thickness on this aspect is relatively minor. B.M. et al. [16,17,18] analyzed the pile-supported reinforced embankment using a plate model, cable-net model and membrane model, and proposed that the shape of the pile cap has a significant effect on the peak strain of reinforcement, and the circular pile cap can substantially reduce the peak strain of reinforcement. The plate model is suitable for the condition of high reinforcement strength, the cable-net model is suitable for the geogrid reinforced cushion, and the membrane model is suitable for the circular pile-cap condition [17]. CAO et al. [19] carried out field-test research on the suspended pile-supported reinforced embankment. It was found that, due to the downward movement of the pile body, the filling load was transferred from the piles to the surrounding soil, resulting in increased load sharing by the intervening soil. The pile-soil stress ratio ranged between 1.5 and 3.7. The membrane effect of the reinforced cushion was found to effectively reduce the differential settlement between the piles and the soil. Moustafa et al. [20] carried out a small-scale model tests on the rigid-pile composite foundation under vertical cyclic loading. The results showed that the cyclic loading improves the load-sharing efficiency of the pile; with the increasing number of loading cycles, the load-bearing efficiency at the pile top increased from 50% to nearly 100%. Displacement of the cushion particles above the pile was small, approximately 0.3 mm, forming a “static soil area”, whereas settlement of the cushion particles between piles was more pronounced, reaching about 6 mm. It should be noted that in the existing literature, cushion materials are predominantly granular, such as graded gravel and crushed stone, whereas studies on cemented materials, including cement-treated soil, as cushion layers remain scarce [21,22,23,24,25]. Nevertheless, the aforementioned research findings still provide valuable insights and serve as a useful reference for the subsequent experimental investigations and numerical analyses presented in this paper. Recent work has further explored rigid-pile-supported embankments with cement-treated load-transfer platforms. Han et al. investigated load-transfer mechanisms of pile-supported platforms with varied fill materials, via trapdoor tests [26]. Liu et al. analyzed settlement and load-redistribution responses of rigid-pile composite foundations subjected to embankment filling [27].

Few studies have systematically investigated the performance of cement-soil cushions combined with prestressed concrete thin-walled pipe piles. The existing literature has extensively characterized geosynthetic-reinforced pile-supported embankments with granular load-transfer platforms, and investigations on PHC-pile composite foundations largely concentrate on gravel cushions or deep-cement-mixing improvement. Although related topics have been reported, full-scale field monitoring, together with quantitative sensitivity analysis, remain scarce for the composite system combining prestressed thin-walled pipe piles and cement-soil cushions. In particular, prior works seldom quantify the relative importance of multiple design variables using a unified evaluation index. To fill this research gap, this work carries out field tests, numerical calculations, and theoretical analysis for this specific composite-foundation solution. The influences of cushion thickness, cement content, reinforcement configuration, pile length, and pile spacing on the bearing-deformation characteristics of the composite foundation are explored, and the relative significance of each influencing factor is quantified. The findings of this study offer experimental data and practical references for the design and application of prestressed concrete thin-walled pipe-pile composite foundations with cement-soil cushions under the site-specific conditions investigated herein.

## 2. Project Profile

**Figure 1:**
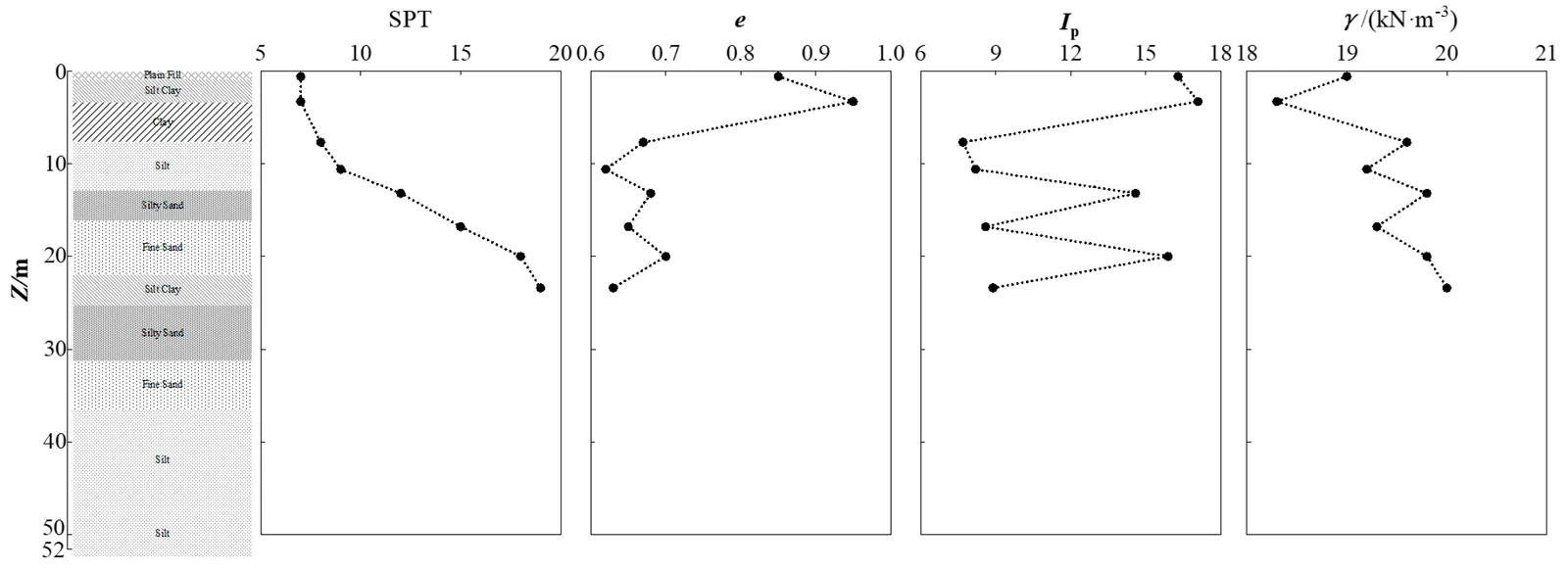
Stratigraphic distribution diagram of test site.

The field-test site was the bridgehead foundation of an expressway in Shandong Province, where the embankment-filling height ranged from 7.4 to 7.8 m. The original design employed gravel-reinforced cushion with prestressed concrete thin-walled pipe piles, which was replaced by a cement-soil reinforced cushion with prestressed concrete thin-walled pipe pile, and the length of pipe pile was 10 m. The soil layer in the field was mainly silt, silty clay and fine sand. The engineering geological survey report showed that the site stratum was divided into 14 engineering geological layers. Among them, the 1-1 layer was plain fill, and layered thickness was 0.6 m, the 2-1 layer was silty clay, and layered thickness was 2.7 m, the 2-2 layer was clay, and layered thickness was 4.4 m, the 2-3 layer was silt, and layered thickness was 2.9 m, the 3-3 layer was silt layer, and layered thickness was 2.6 m, the 3-4 layer was silty sand layer, and layered thickness was 3.6 m, the 3-5 layer was fine-sand layer, and layered thickness was 6.2 m, the 4-1 layer was silty clay layer, and layered thickness was 3.4 m, the 4-5 layer was silty sand layer, and layered thickness was 6.4 m, and the 4-5 layer was fine-sand layer, and layered thickness was 5.6 m. The 5-3 layer was the silt layer, and the layer thickness was 11.6 m, the 6-3 layer was the silt layer, and the average thickness was 5.0 m, and the 6-4 layer was the silt layer, which was not penetrated by geological exploration. According to the collected regional geological data, geological survey, and exploration data, no evidence of fault or fracture development was found at the site. The site strata consist of loose accumulations from the field Quaternary Holocene and Upper Pleistocene series. The adverse geological conditions and problematic soils identified at the site mainly included seismic liquefaction and plain fill deposits. With appropriate engineering measures, the impact on the pile foundation was considered to be minor, and the overall foundation stability was satisfactory. The field stratigraphic distribution diagram is shown in Figure 1.

The field-test program primarily focused on two variables: cement-soil cushion thickness and the number of geogrid reinforcement layers. For the instrumented test section, prestressed concrete thin-walled pipe piles with an outer diameter of 400 mm, wall thickness of 60 mm and pile length of 10 m were adopted. The cement-soil cushion was mixed on-site and compacted in layers. Sampling and compressive-strength tests were performed, to control its construction quality.

**Figure 2:**
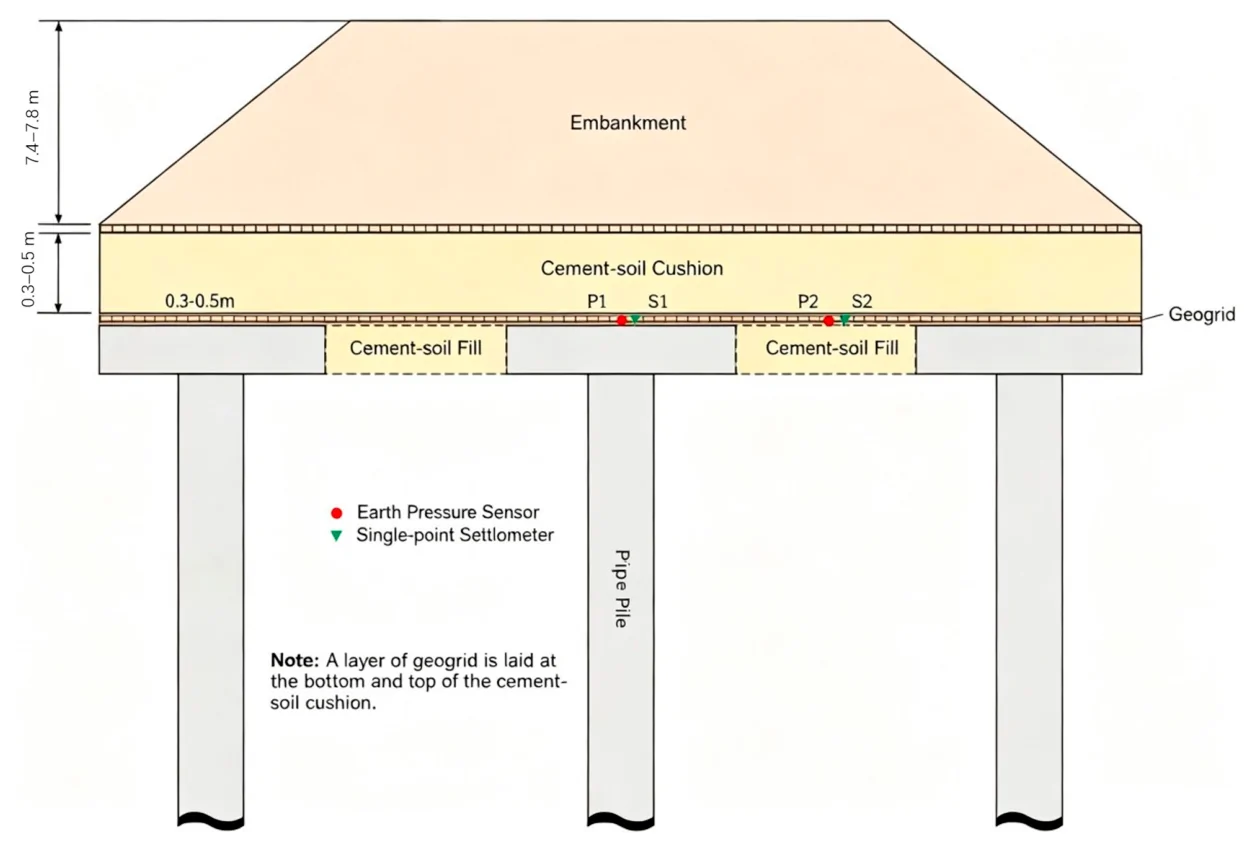
Schematic diagram of sensor arrangement for field-test scheme.

Three cushion-thickness levels (300 mm, 400 mm and 500 mm) were designed for the cement-soil cushion. Regarding geogrid-reinforcement layers, two working conditions with single-layer reinforcement at the cushion bottom and double-layer reinforcement at both the top and bottom of the cushion were arranged (both under a 300 mm thick cushion). A control group with a gravel cushion reinforced by one geogrid layer at the bottom was also set up. The detailed field-test scheme is summarized in Table 1, and the sensor-layout schematic is presented in Figure 2.

Earth-pressure cells and settlement sensors were adopted for field monitoring. All sensors were strictly calibrated in the laboratory before field installation. The sensors were embedded at designated positions including the pile top and inter-pile soil layer to capture real-time settlement and earth-pressure responses. Data acquisition was performed once per day throughout the entire filling construction period. The nominal accuracy of all monitoring sensors was better than 0.5% full scale, ensuring the reliability of the measured data.

## 3. Numerical Calculations

A total of seven influencing factors were considered in the numerical simulations, as detailed in Table 2. The cement content mainly considered the influence of the change in the cushion stiffness on the bearing and deformation characteristics of the composite foundation, and the rest also considered the cushion thickness, the number of reinforcement layers, the reinforcement position, the pile-cap size, the pile length and the pile spacing. The above six factors were systematically investigated for their effects on the composite foundation performance. According to the influencing factors in Table 2, the specific calculation conditions were determined, which are summarized in Table 3, with a total of 17 groups of working conditions.

A three-dimensional finite-element model was established, using the commercial software ABAQUS 2022. The modeling procedure comprised seven main steps: part creation, property assignment, assembly, analysis step configuration, interaction definition, boundary condition and load application, and mesh generation. The detailed modeling process is described as follows.

(1) Components.

Part creation: the model includes foundation, pile, cushion, reinforcement layer and embankment, and the calculation is simplified by the 1/2 symmetric model. The corresponding components of each part are established according to the simplified model parameters.

(2) Attribute definition.

Property assignment: the material model is assigned to each component: the Mohr–Coulomb elastic–plastic model is used for the foundation soil and the embankment fill, and the elastic model is used for the pile. Homogeneous materials (solid components) are defined and assigned to the corresponding components through the “cross-section” tool.

(3) Assembly.

Assembly: the independent components are combined into a complete model, and the components are tightly assembled into a whole, from bottom to top, according to the order of the simplified model-parameter table. Among them, surface-to-surface contact is defined between pipe piles and the surrounding foundation soil, and the reinforcement (geogrid) is embedded in the cushion, via embedded-element constraint.

(4) Analysis-step setting.

Analysis-step configuration: multiple analysis steps need to be set when setting the analysis step. In the initial analysis step, the initial boundary and predefined field of the model are set, and the initial state is established. The in situ stress analysis step calculates the initial stress field caused by self-weight by applying gravity to ensure the self-weight balance of the model. The deformation and stress response of the structure under static loading are simulated by applying embankment load in the static general-analysis step.

(5) Definition of interaction.

Surface-to-surface contact pairs are established only between pile-foundation soil interfaces. The geogrid is incorporated into the cushion via embedded-element constraint, so no additional contact pairs are assigned between geogrid and cushion or embankment materials. For the pile-soil contact pairs, hard contact is assigned in the normal direction, while the penalty function algorithm is adopted for tangential behavior. The penetration tolerance is set to 1–5% of the component size, to avoid numerical penetration. For pile–soil interfaces, the interface friction coefficient is set to 0.7 times the internal friction angle of the adjacent soil layer, to govern tangential shear transfer. For native foundation soils, cohesion, friction angle and dilation angle originate from laboratory tests on site-investigation specimens. Embankment-fill parameters are obtained from material test data of the same highway project. Dilation angles are taken as one-half of the corresponding friction angles, according to common geotechnical finite-element practice.

(6) Boundary conditions.

Boundary conditions: the bottom of the model is fixed, the lateral displacement is limited, and the semi-infinite foundation is simulated.

(7) Grid division.

Mesh generation: the eight-node hexahedron (C3D8R) solid element is used for foundation soil, embankment and pile, and the pile top, pile body and reinforcement are locally densified. The divided grid is mainly quadrilateral, and the curvature is controlled at 0.1. The free technology is adopted, and the mapping grid is used in the appropriate position.

A three-dimensional numerical model was established through the coordinated configuration of seven modules: part creation, property assignment, assembly, analysis-step setup, interaction definition, boundary condition and load application, and mesh generation. The model reproduces the geometric configuration of the embankment and composite foundation, and adopts realistic material properties and in situ stress conditions. Local mesh refinement is implemented near the pile, cement-soil cushion, and geogrid regions with high stress gradients, while coarser meshes are adopted in areas far from the composite foundation. This mesh arrangement follows common geotechnical finite-element practice, and is calibrated against field-monitoring data.

**Figure 3:**
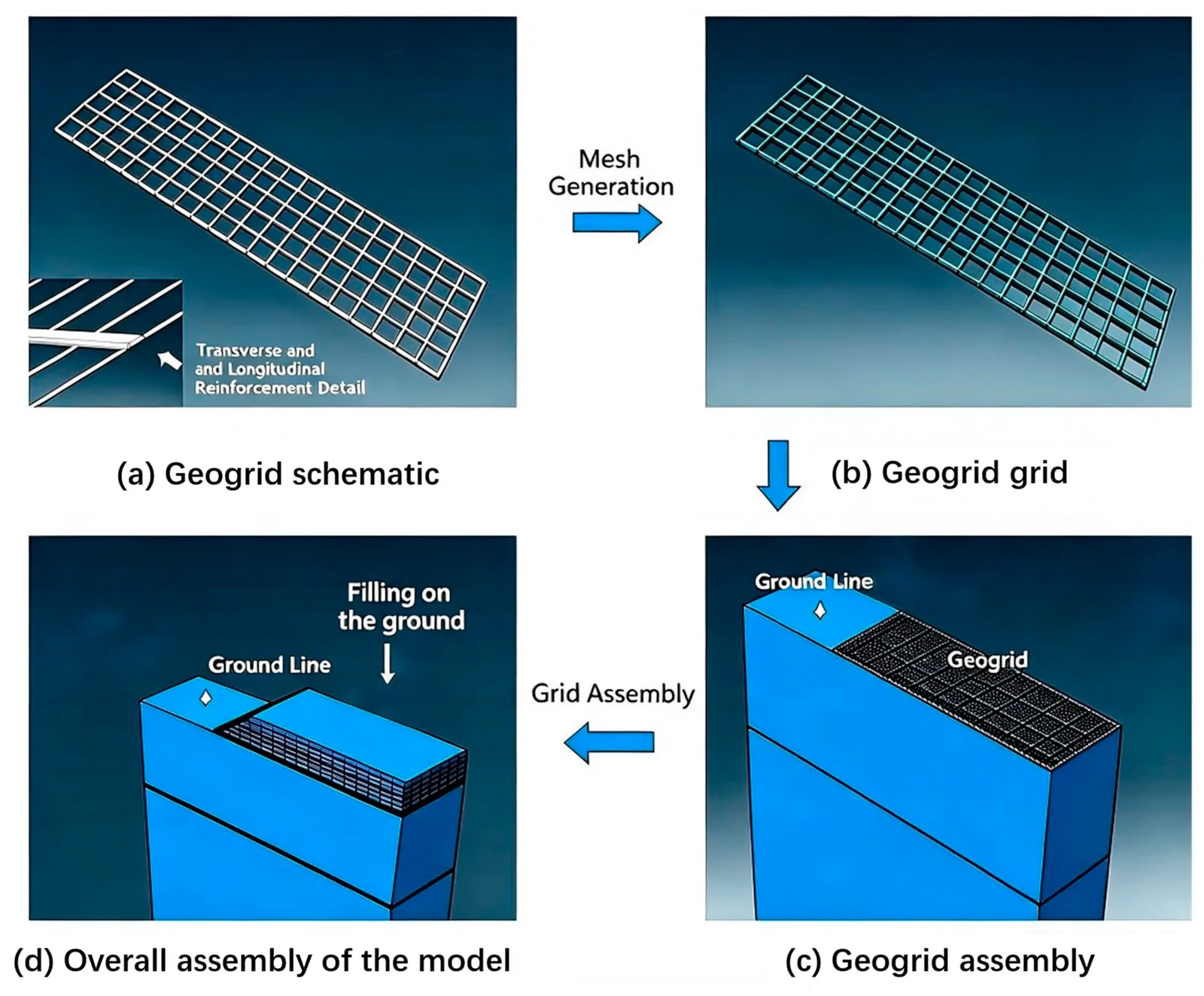
Geogrid-modeling flow chart.

**Figure 4:**
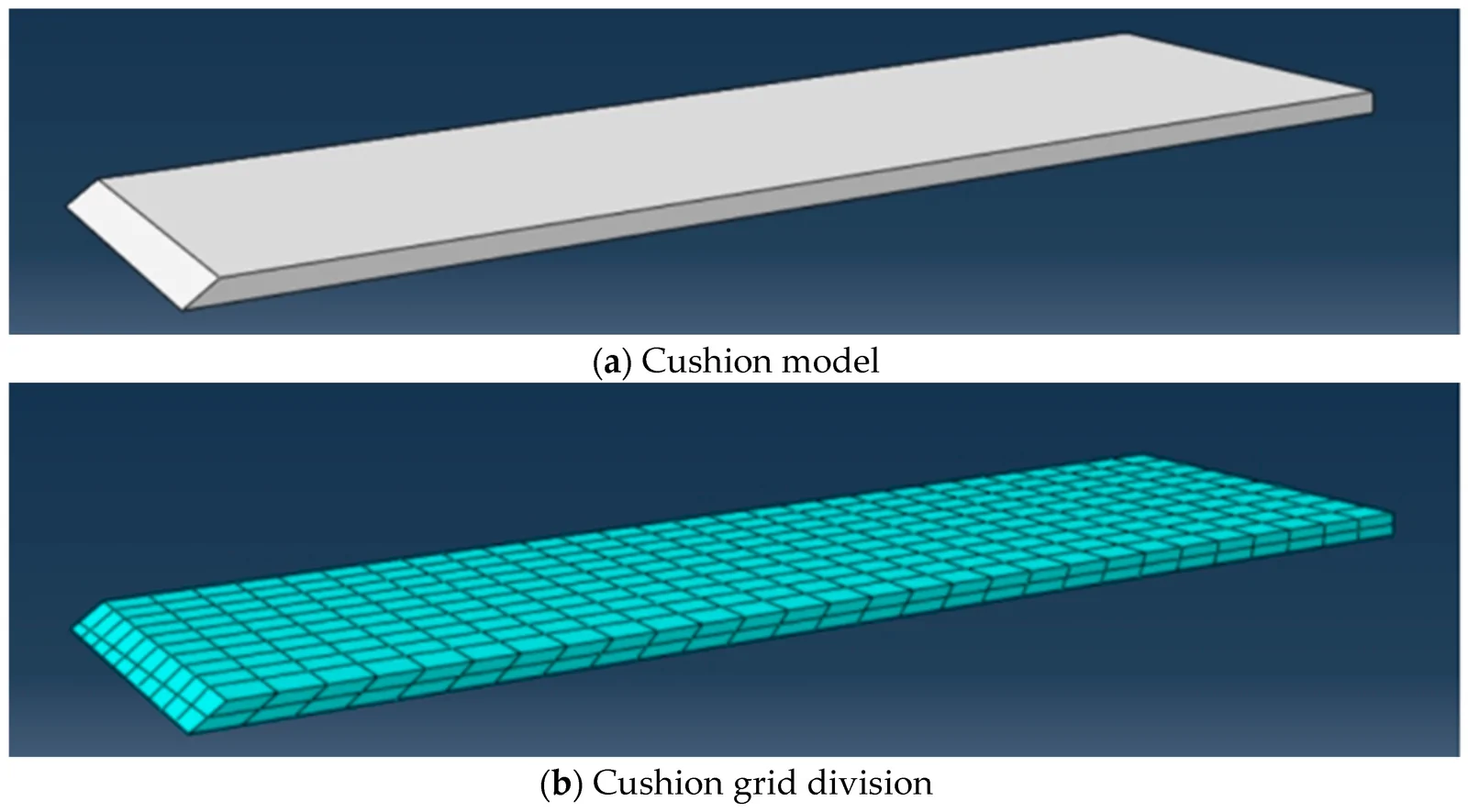
Establishment of cushion model.

In order to accurately simulate the actual working conditions, the ABAQUS finite-element software was used to complete the three-dimensional geometric modeling based on the actual size, material properties and laying form of the geogrid, and the geogrid was assembled with the surrounding soil and the upper and lower structures into an overall calculation model. The material parameters of the geogrid are summarized in Table 4. The modeling processes for the geogrid and the cushion are illustrated in Figure 3 and Figure 4, respectively.

The cement-treated soil cushion acts as a critical component for load transfer inside the composite foundation. P.O 42.5 ordinary Portland cement, consistent with that used in field tests, was adopted for cushion preparation. Cement dosage is defined as the mass ratio of cement to dry base soil, and the base soil is low-liquid-limit silt sampled from the Jide Expressway test site. Specimens were mixed at optimum water content and cured for 28 d before mechanical property characterization. The complete Mohr–Coulomb constitutive parameters of cement-treated soil under cement dosages of 4%, 6% and 8% are listed in Table 5.

**Figure 5:**
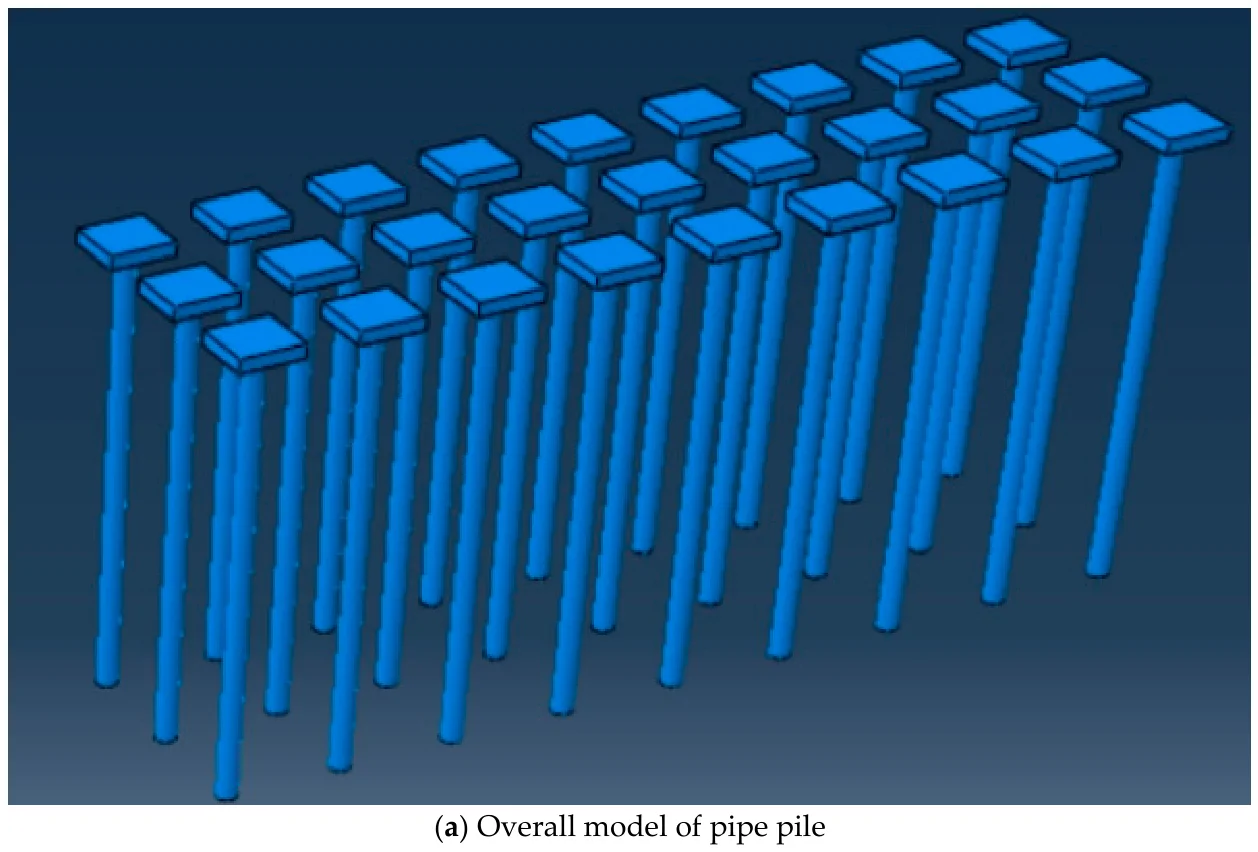

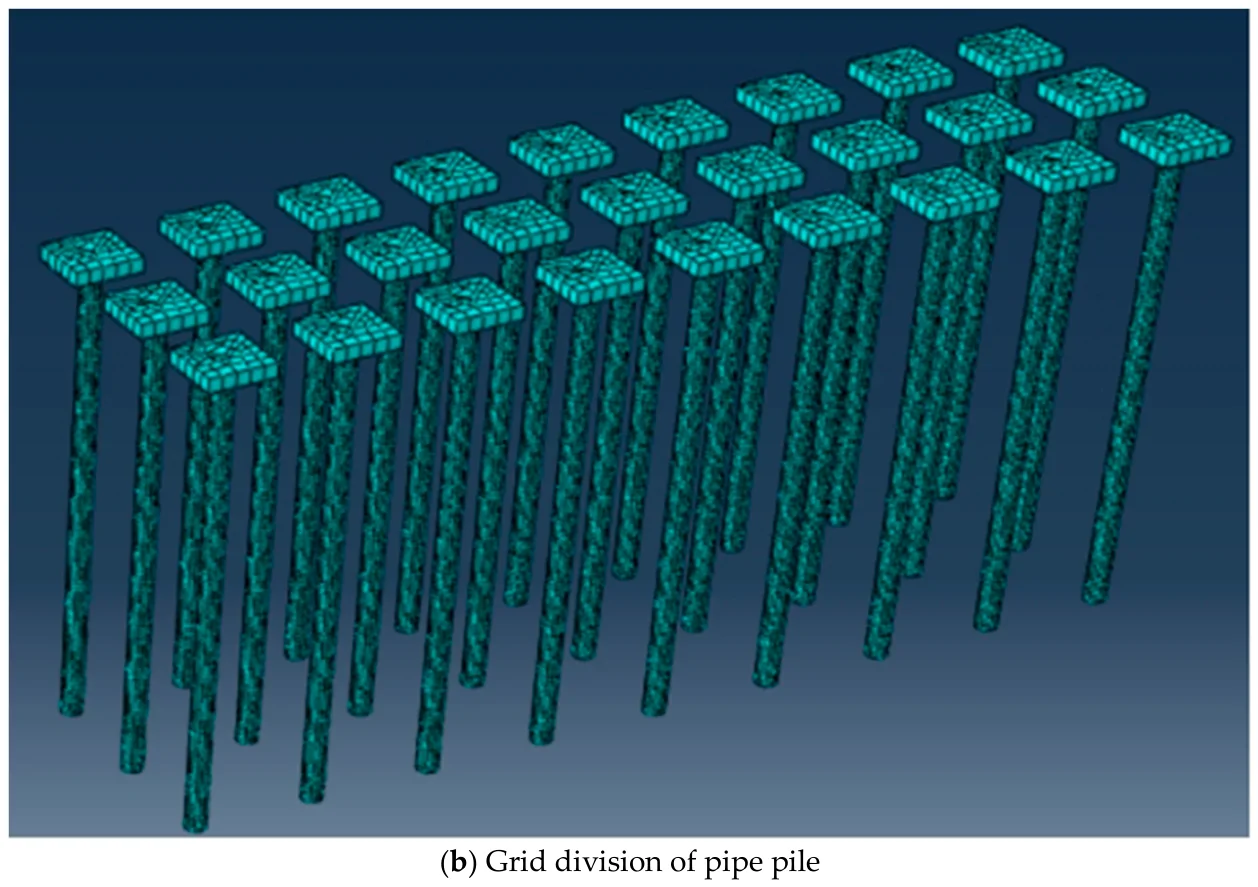
Model of pipe pile.

The elastic model is adopted for the pile body, and the calculation parameters of the pile body are shown in Table 6. The pile-body model and grid division are shown in Figure 5.

**Figure 6:**
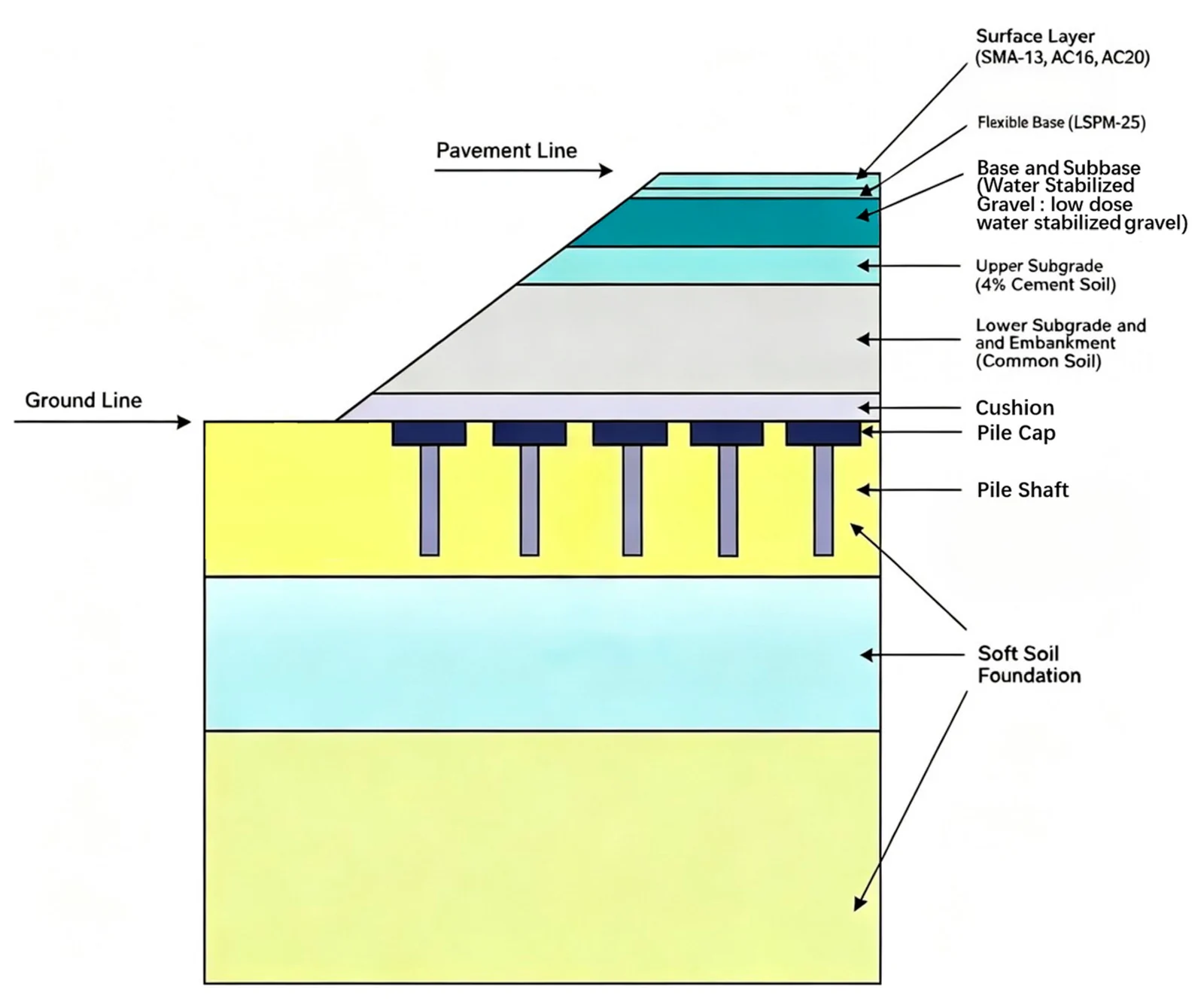
Structure diagram of calculation model.

**Figure 7:**
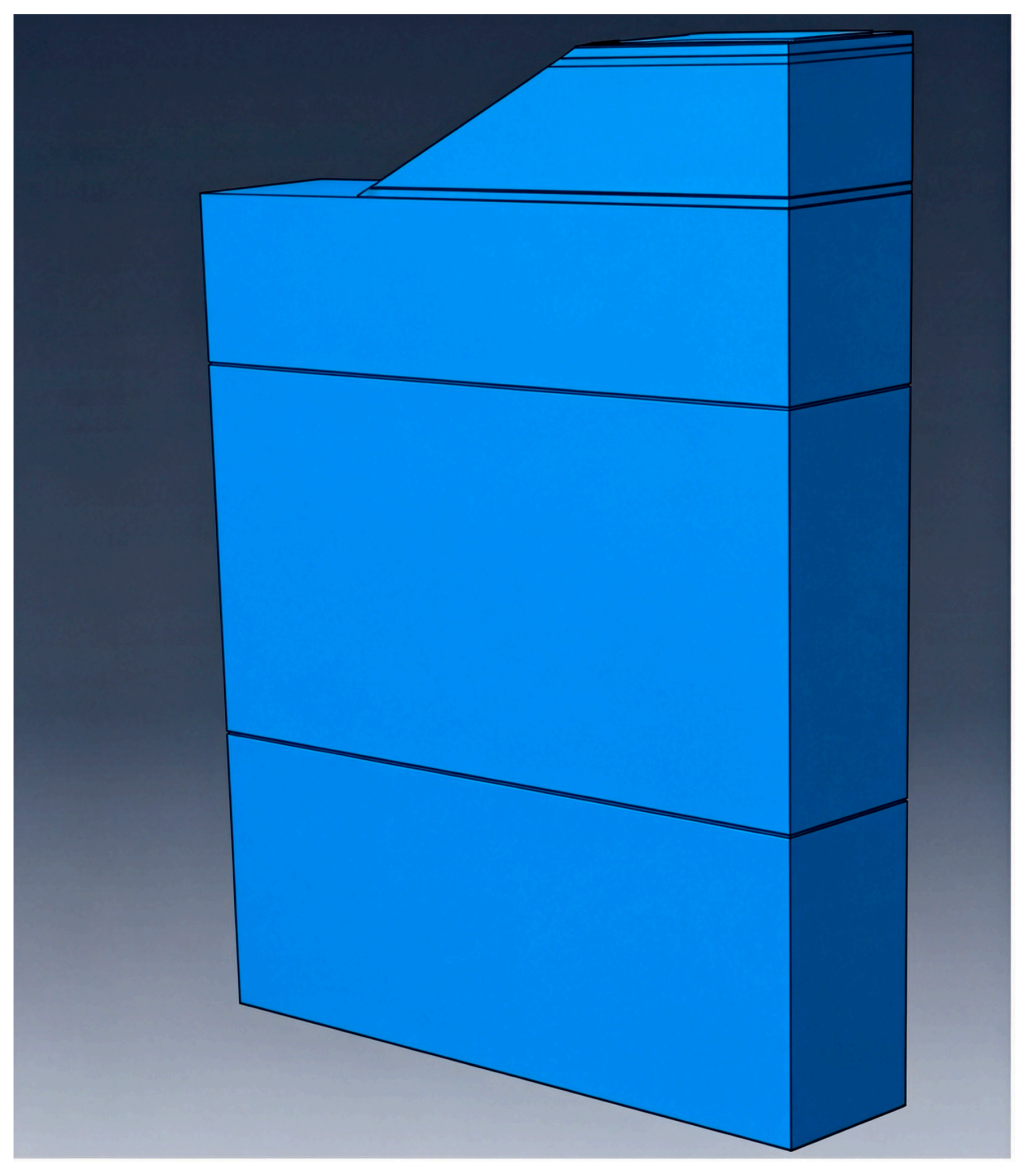
Calculation model.

The calculation model of the cement-soil reinforced cushion (gravel cushion) of prestressed concrete pipe pile is shown in Figure 6. The calculation model is shown in Figure 7 and the soil parameters of the model are shown in Table 7. Cohesion and friction angle for native foundation soils come from laboratory tests carried out for this project. Mohr–Coulomb parameters of embankment fill are taken from test data of comparable fill soils on the same highway site. Dilation angles are set to half of the friction angles, following standard geotechnical finite-element practice.

In the calculation process, the embankment-filling process is simulated. One layer of filling soil is set every 0.5 m, to simulate the on-site filling construction process. The embankment is piled up to a height of 6 m, and then the filling is stopped. The relevant geotechnical input parameters are listed in Table 8, below.

## 4. Analysis of Effect

### 4.1. Field-Test Data and Model Verification

**Figure 8:**
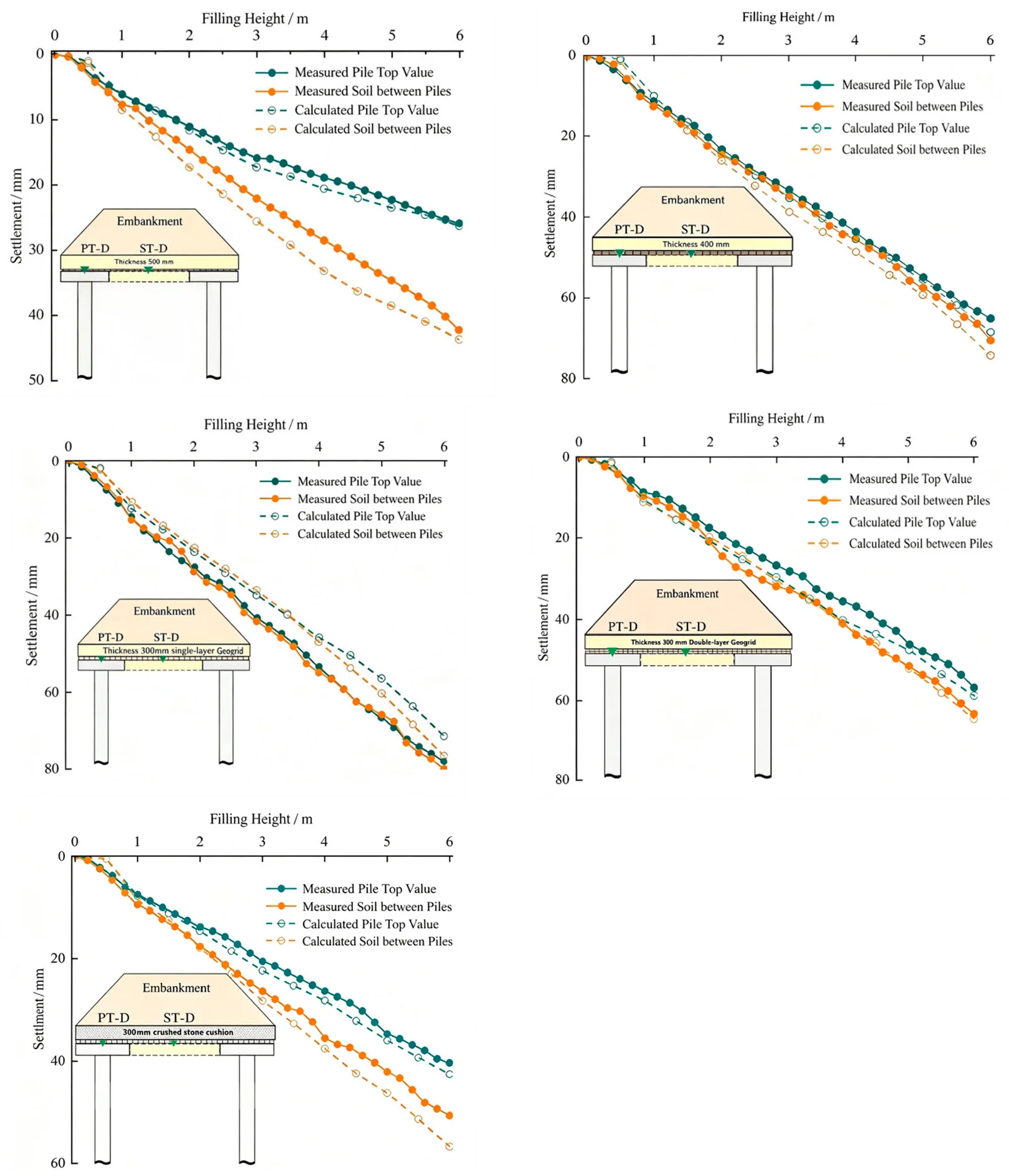
Comparison of calculated and measured settlement deformation values.

**Figure 9:**
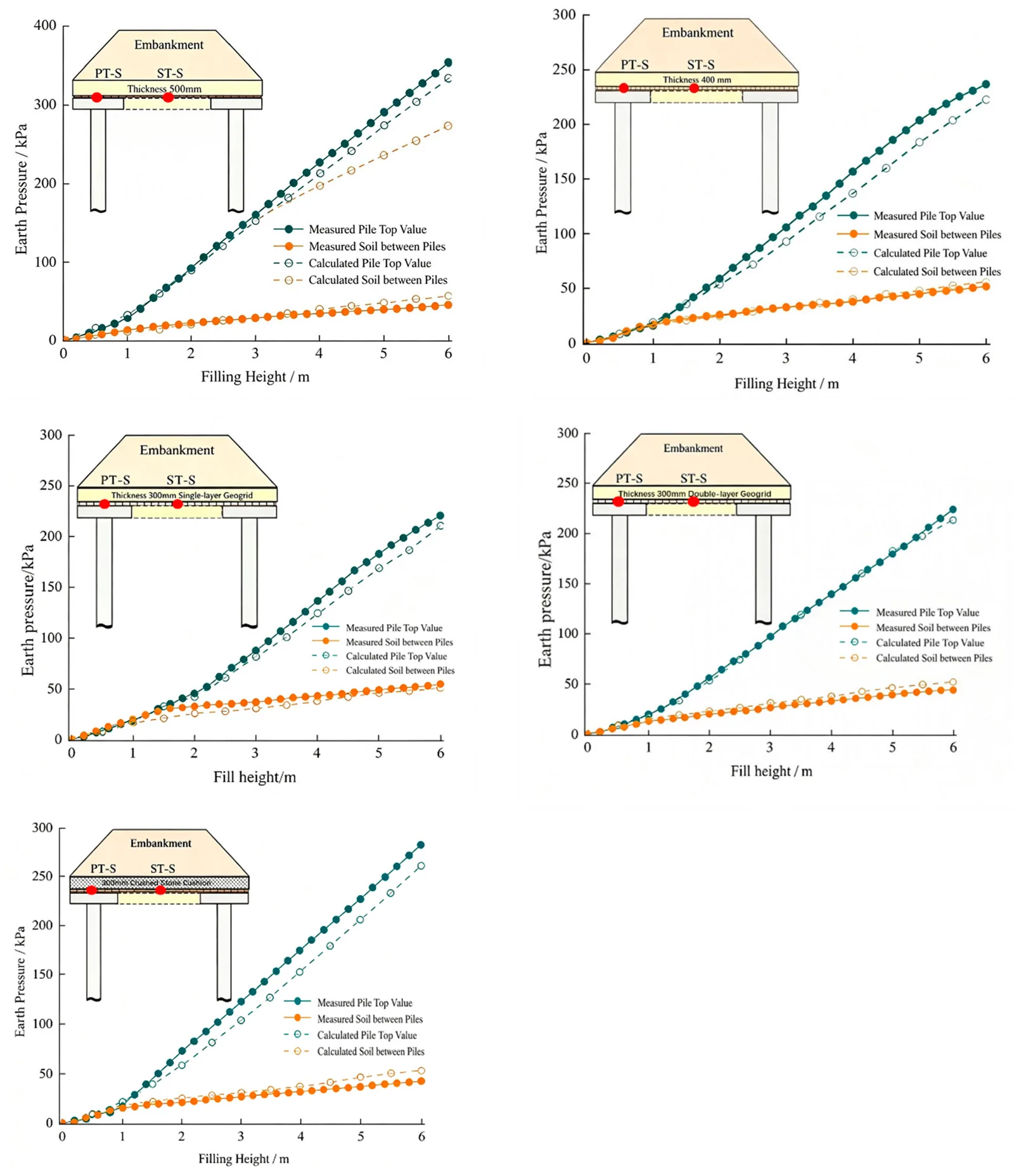
Comparison of calculated value and measured value of soil pressure.

The numerical-analysis model established in this paper is used to compare the calculation results with the measured numerical results for the five groups of working conditions in the field test. The comparison between the calculated results and the measured results is shown in Figure 8 and Figure 9. From the comparative analysis of the results, it can be seen that the numerical-calculation results are basically consistent with the measured results, which can describe the law of the test results more accurately, and prove that the numerical-calculation model established in this paper is reasonable and reliable.

It should be noted that the field monitoring test was implemented during the first-stage embankment filling, up to 6 m. Although the final designed embankment height of this bridgehead section ranges from 7.4 m to 7.8 m, the fill above 6 m had not been constructed at the time of field testing. The field monitoring campaign ended upon completion of this first-stage filling. Accordingly, all simulation-measurement comparisons illustrated in Figure 8 and Figure 9 and Table 8 refer specifically to the 0–6 m first-stage filling construction stage.

From the data analysis in the figure, it is concluded that the smaller the thickness of the cement-soil cushion, the larger the settlement deformation of the pile top and the soil between the piles. The double-layer geogrid is better than the single-layer geogrid at controlling the settlement, and the pile-soil settlement is reduced by about 20%. This comparison corresponds to two complete foundation configurations, namely a 500 mm thick cement-soil reinforced cushion and a 300 mm thick gravel-reinforced cushion. Variations occur in both cushion material and cushion thickness, simultaneously. The observed difference in settlement-control performance therefore reflects the combined influence of material property and cushion thickness. For this set of configurations, the pile-top settlement decreases from 40 mm to 25 mm (a reduction of approximately 37%), and the inter-pile soil settlement decreases from 52 mm to 42 mm (a reduction of approximately 19%). The greater the thickness of the cement-soil cushion, the stronger the ability to resist deformation, which can make the pile bear more overlying load, reduce the load borne by the soil between piles, give full play to the advantages of the high bearing capacity of prestressed concrete pipe piles, and reduce the total settlement of the composite foundation. The greater the thickness of the cushion, the greater the differential settlement of pile and soil, and the differential settlement of pile and soil reflects the size of the soil-arching effect. The larger the thickness of the cushion, the higher the bearing capacity of the pile, and the higher the degree of soil-arching effect, the greater the differential settlement of the pile and soil.

Corresponding to the settlement curve, the greater the thickness of the cushion, the greater the load on the top of the pile, and the soil pressure on the top of the pile shows 500 mm cement-soil-reinforced cushion > 300 mm gravel-reinforced cushion > 400 mm cement-soil-reinforced cushion > 300 mm cement-soil-double-reinforced cushion > 300 mm cement-soil-reinforced cushion. With the increase in the thickness of the cushion, the stiffness of the cushion increases, and the ability to resist deformation increases, so that the pile bears more load. The bearing mechanism of the prestressed concrete pipe-pile composite foundation mainly includes the pile-cap effect, soil-arching effect and membrane effect. Among them, the pile-cap effect and soil-arching effect are the main effects, and the membrane effect is supplemented. When the cement-soil-reinforced cushion is used to replace the gravel-reinforced cushion, because the cement soil is a cohesive material, the deformation adjustment ability of the cement-soil cushion is weaker than that of the gravel cushion, but it is beneficial to the flexible load of the embankment. And the cement-soil-reinforced cushion exerts the load-redistribution platform effect, covering the surface of the composite foundation, which enhances the integrity of the composite foundation. For the concrete pipe-pile composite foundation, when the thickness or stiffness reaches a certain value, it has a cap effect on the composite-foundation bearing, which can further transfer the upper-embankment load to the pile top, increase the load borne by the pile body, and reduce the soil load between the piles. This reduces the load transfer to the deep soil, reducing the compression deformation of the underlying soil, and reducing the settlement of the foundation.

Table 9 summarizes the measured and simulated settlement and earth-pressure values at representative filling heights for Case 1. Quantitative indicators, including R^2^, RMSE, MAPE and maximum relative error are calculated for Case 1 only, as listed in Table 9. Visual comparison of Figure 8 and Figure 9 indicates that good agreement between numerical predictions and field-monitoring data is achieved for all five test cases.

### 4.2. Analysis of Different Influencing Factors

#### 4.2.1. Cement Content

**Figure 10:**
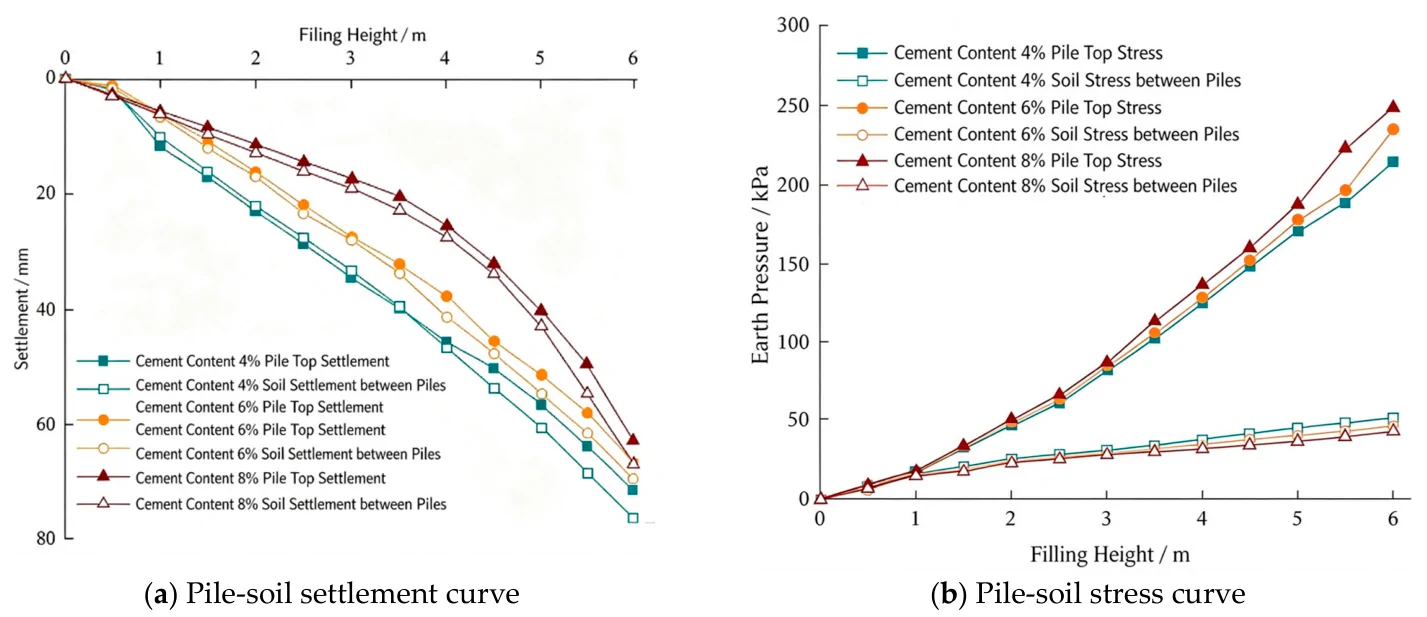
Test results of cemen- soil cushion with different cement content.

In order to compare the influence of cushions with different cement contents on the bearing-deformation characteristics of the composite foundation, numerical simulations were carried out for cement contents of 4%, 6% and 8%. The settlement-deformation results are shown in Figure 10a, and the pile-soil stress results are presented in Figure 10b. With the increase in cement content, the settlement values of pile top and inter-pile soil decrease gradually, while the earth pressure on the pile top increases. Higher cement content improves the strength and stiffness of the cement-soil cushion, enhancing its rigid-cushion effect and enabling piles to carry more load. In practical engineering, appropriately increasing the cement content of the cushion can improve cushion strength, give full play to pile-bearing capacity, and reduce the settlement of the composite foundation. The change in pile load-sharing ratio further demonstrates the plate-like load-distribution effect of the cement-soil cushion. As cement content grows and cushion stiffness increases, embankment loads are redistributed toward pile tops, leading to an elevated pile load-sharing ratio. This observation reveals that the stiffer cement-soil cushion acts as a relatively stiff load-transfer platform, and promotes the development of soil arching between piles.

#### 4.2.2. Thickness of Cement-Soil Cushion

**Figure 11:**
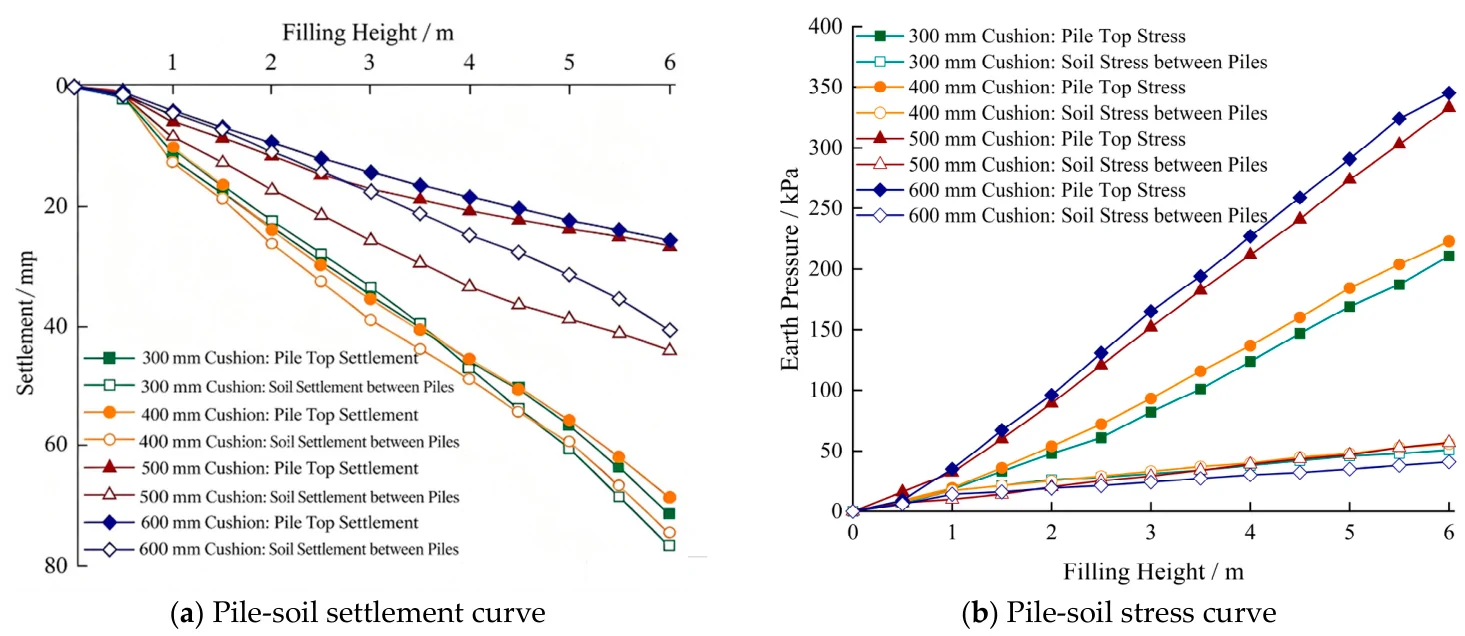
Results of different cement-soil cushion thickness.

The settlement results of different cushion thicknesses are shown in Figure 11a, and the calculation results of pile-soil stress are shown in Figure 11b. The results show that the thickness of the cushion has an important influence on the bearing and deformation characteristics of the pipe-pile composite foundation. The thickness of the cushion is large, the settlement of pile and soil becomes smaller, and the stress of the pile increases. When the thickness of the cushion increases from 300 mm to 400 mm, the settlement of the pile top and the soil between the piles does not change much, and the increase in the pile pressure is not obvious. When the thickness of the cushion increases from 400 mm to 500 mm, the settlement deformation decreases by an obvious amount, and the pile-soil stress also increases greatly. As the cushion thickness increases from 400 mm to 500 mm, the settlement of the pile top decreases from 68.5 mm to 26.3 mm, a reduction of 61.6%. When the cushion thickness increases from 500 mm to 600 mm, the pile-soil settlement and pile-soil stress do not change much. This shows that the cement-soil reinforced cushion needs to reach a certain thickness to give full play to the rigid-cushion effect. When this apparent diminishing-return thickness is reached, the effect of increasing the cushion thickness on settlement and pile-soil stress is not obvious.

#### 4.2.3. Reinforcement Setting

**Figure 12:**
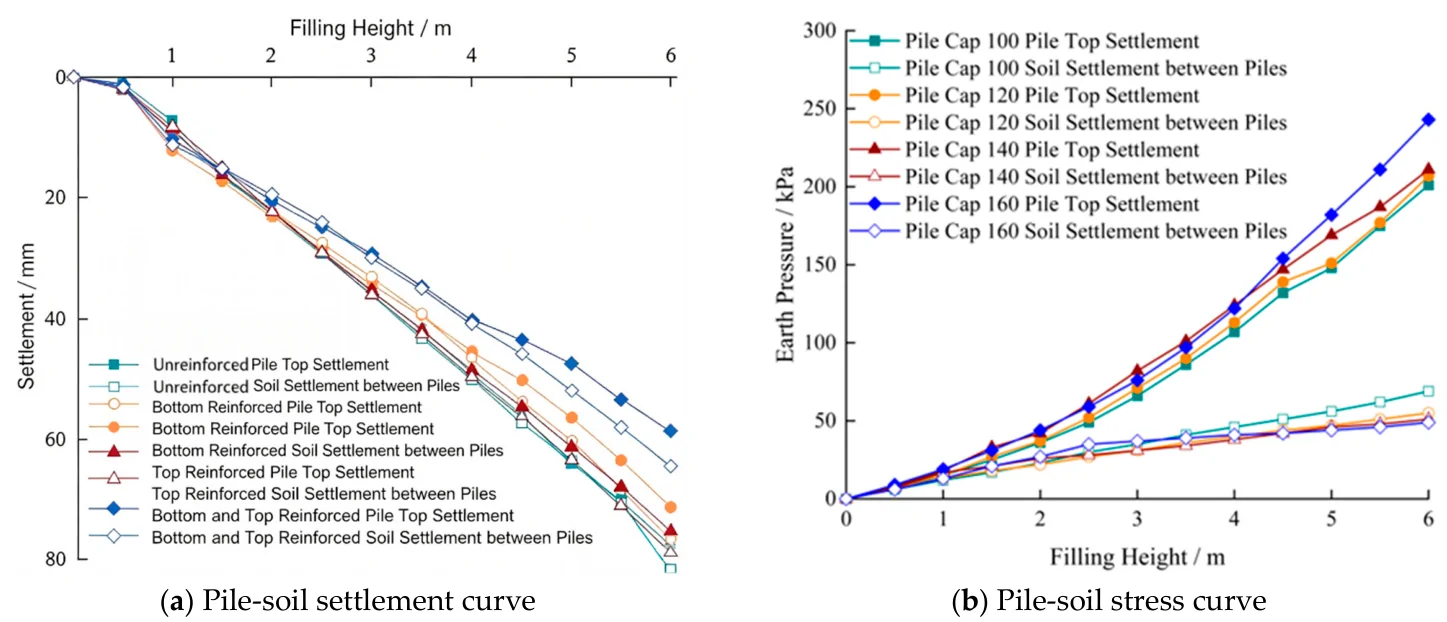
Different reinforcement-setting results.

The settlement and pile-soil stress calculation results of different reinforcement numbers and positions are shown in Figure 12a and Figure 12b, respectively. The effect of double-layer reinforcement is better than that of single-layer reinforcement and no reinforcement. Under the condition of single-layer reinforcement, the reinforcement effect of bottom reinforcement is better than that of top reinforcement. Compared with the single-layer reinforcement at the bottom, the settlement of the double-layer reinforcement is reduced by 12.7 mm. Considering the convenience and economy of construction, the single-layer reinforcement cushion at the bottom can be selected. Considering the settlement-control effect, the double-layer reinforcement cushion at the top and bottom can be selected.

#### 4.2.4. Size of Pile Cap

**Figure 13:**
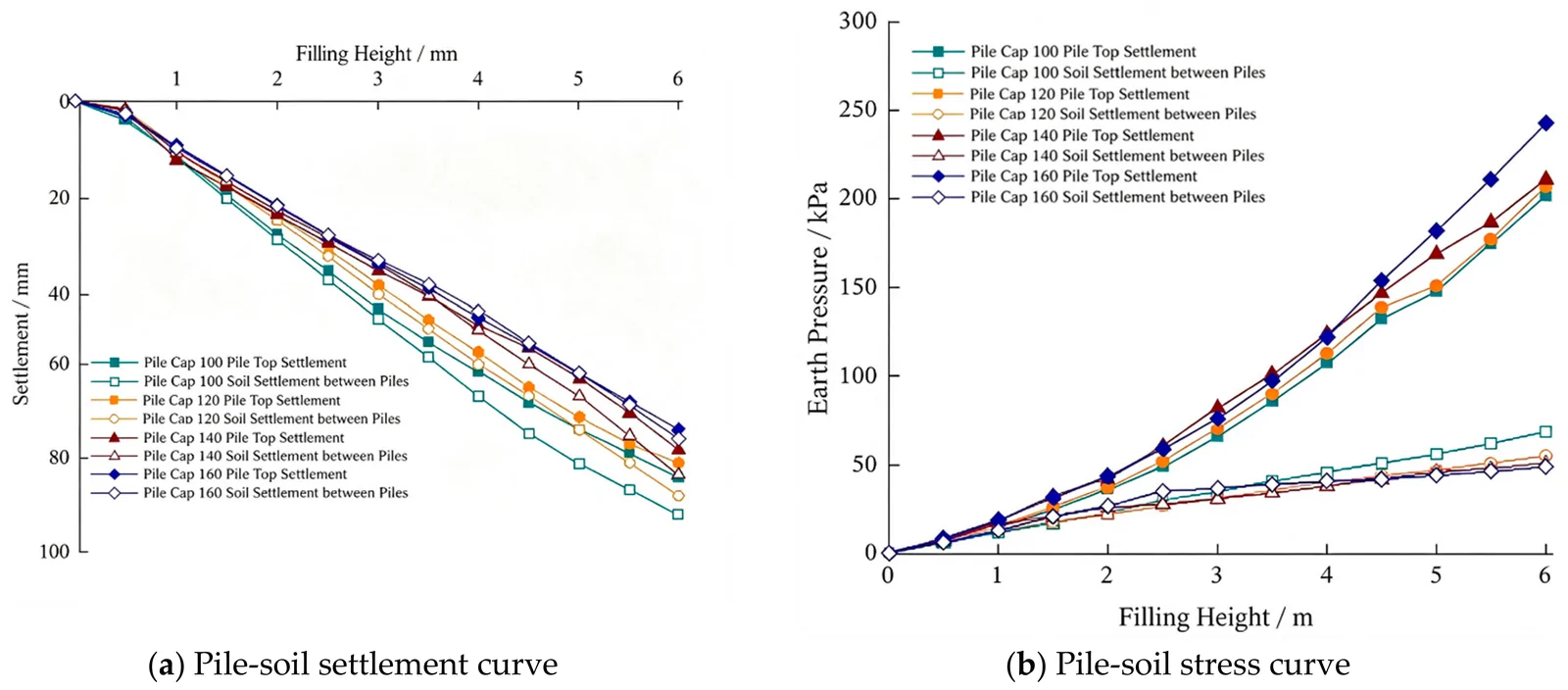
Results of different pile-cap sizes.

The calculation results of pile-soil settlement and stress under different pile-cap sizes are shown in Figure 13a,b. The larger the pile-cap size, the greater the pile-top stress, the smaller the settlement, the larger the pile-cap size, and the more obvious the pile-cap effect and soil-arching effect. The pile-cap size increases from 100 mm to 160 mm, and the pile-top settlement value decreases by 10 mm. From an economic point of view, it can be considered to appropriately reduce the pile-cap size, and save the project cost.

#### 4.2.5. Length of Pile

**Figure 14:**
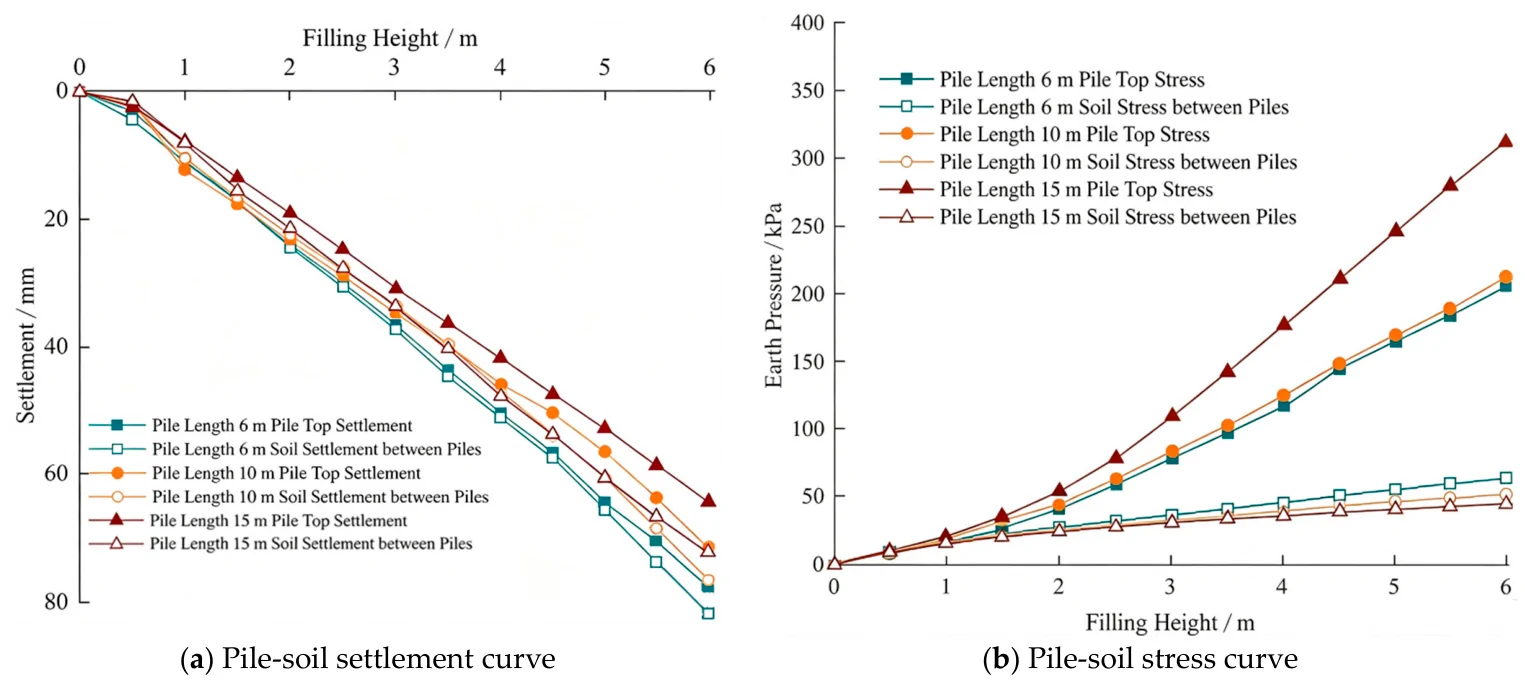
Results of different pile lengths.

The calculation results of pile-soil settlement and stress under different pile lengths are shown in Figure 14a,b. With the increase in pile length, the settlement of the pile-soil decreases and the stress of the pile top increases. The settlement of the pile top under the condition of 15 m pile length is 13.6 mm lower than that under the condition of 6 m pile length, which is reduced by 21.3%. The settlement of the pile top under the condition of 10 m pile length is 6.3 mm less than that under the condition of 6 m pile length, which is reduced by 8.8%. Compared with the proportion of pile-length increase, its influence on settlement control is not very significant. The determination of pile length should be based on bearing-capacity requirements and passing through the soft-soil layer, as the main design basis.

#### 4.2.6. Pile Spacing

**Figure 15:**
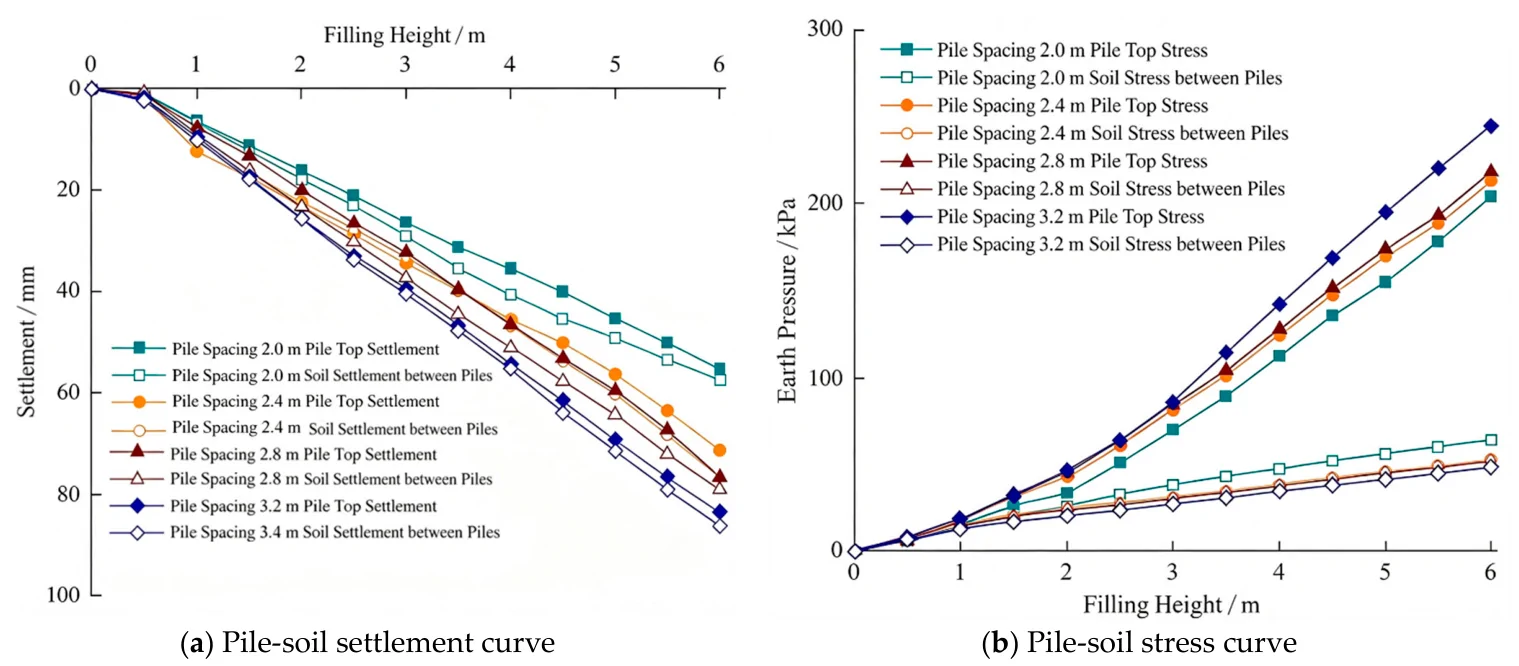
Results of different pile spacing.

The calculation results of pile-soil settlement and stress under different pile-spacing conditions are shown in Figure 15a,b. Pile spacing has a great influence on pile-soil settlement and bearing characteristics. With the decrease in pile spacing, the load borne by a single pile body decreases, and the pile-soil stress ratio decreases. However, due to the small pile spacing, the total load borne by the pile body is higher, and the pile-soil settlement is smaller under the unit treatment area. With the increase in pile spacing, the reinforcement area of the single pile becomes larger, and the higher the load borne by the single pile, the greater the pile-soil stress ratio. However, under the unit treatment area, due to the large pile spacing and the small number of piles, the total load ratio borne by the pile is low, and the pile-soil settlement is larger.

Pile spacing and pile-cap size are key technical parameters for the design of prestressed concrete thin-walled pipe-pile composite foundations. In this study, a total settlement of 80 mm was adopted as an engineering serviceability-oriented threshold, to determine the maximum pile spacing and minimum pile-cap size for the investigated working conditions. Under this threshold, the corresponding pile spacing is 2.8 m and the pile-cap size is 1.4 m. For the cases examined herein, this yields a pile-cap-size-to-pile-spacing ratio of 0.5 and a pile-cap area replacement ratio of 25%. This recommended ratio applies only to the geological conditions and foundation configurations considered in this study, and cannot be generalized to other ground conditions without further verification.

### 4.3. Analysis of the Influence Degree of Different Factors

In order to evaluate the influence degree of different influencing factors on the settlement and deformation characteristics of pipe-pile composite foundation, the DOI (Degree of Influence) parameter is introduced to evaluate the influence degree of different factors on the settlement and deformation of composite foundation. The calculation of DOI parameters is shown in Formula (1):

(1) $$\mathrm{DOI}=\frac{\left|\alpha_{\max}-\alpha_{\min}\right|}{\left(\alpha_{\max}+\alpha_{\min}\right)/2}$$

In the formula, *α*_max_ is the maximum settlement under the influence of a factor, and *α*_min_ is the minimum settlement under the influence of a factor.

**Figure 16:**
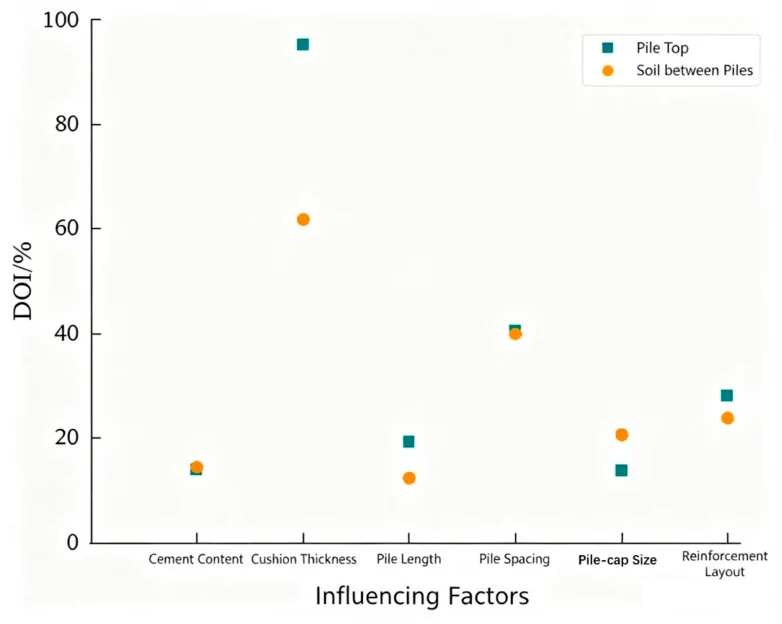
DOI results of different factors on settlement.

The higher the DOI value, the more significant the effect of this factor on settlement. The DOI calculation results of the influence of different factors on the settlement of composite foundation are shown in Figure 16.

It can be seen from the above results that cushion thickness exerts the most significant influence on settlement, with a DOI of approximately 95% for pile-top settlement and 61% for inter-pile soil settlement. Pile spacing is the second most influential factor, with a DOI of around 40% for both pile top and inter-pile soil settlement. Cement content shows the weakest influence, and the DOI values of the remaining factors are below 30%. Considering the degree of influence (DOI), construction-period settlement and field-test results, a 400 mm cushion thickness and 25% pile-cap area-replacement ratio yield satisfactory performance under the conditions of this study. Pile length should be designed primarily to meet bearing-capacity requirements and penetrate through soft-soil layers. The above empirical values are only valid for the geological and loading conditions examined in this work, and should not be directly adopted for other projects without further evaluation. This factor ranking is only applicable to the parameter ranges considered in this study, and cannot be generalized to other uninvestigated working conditions. Future research may adopt normalized sensitivity indices or ANOVA factorial analysis, to achieve more robust sensitivity assessment.

## 5. Conclusions

Based on full-scale field-test measurements, this paper investigates the bearing-deformation performance of prestressed concrete thin-walled pipe-pile composite foundations with cement-soil cushions, via numerical simulation. Special attention is paid to revealing the variation rules of pile-soil stress and settlement, as well as quantifying the relative influence magnitude of key design factors. The main findings are summarized as follows:

(1) The pile-soil settlement decreases with the increase in cement content, cushion thickness, pile-cap size and pile length, and increases with the increase in pile spacing. The pile-soil stress ratio increases with the increase incement content, cushion thickness, pile-cap size, pile length and pile spacing.

(2) The thickness of the cushion is an important factor affecting the stress-change and settlement-deformation characteristics of the pile and soil. The thickness of the cushion has the most significant influence on the stress and settlement of the pile and soil, followed by the pile spacing, and the cement content has the least influence. Increasing the thickness of the cushion is an effective measure to control the settlement of the pipe-pile cement-soil cushion-composite foundation. There is an apparent diminishing-return thickness for the cushion. When the cushion thickness exceeds this value, the effect of increasing cushion thickness on settlement control is not obvious.

(3) From the point of view of settlement-control effect, the effect of double-layer reinforced cushion is better than that of single-layer reinforced and unreinforced cushion, and the effect of single-layer bottom reinforcement is better than that of single-layer top reinforcement. The more layers of reinforcement layer there are, the higher the proportion of pile-bearing load, and the horizontal constraint effect of the reinforcement layer on the cushion is stronger when it is located at the bottom of the cushion, which is more conducive to the role of the rigid-cushion effect of cement soil.

(4) The pile-cap size and pile spacing are important design parameters that affect the bearing and deformation characteristics of pipe-pile composite foundation. According to the calculation results using the 80 mm total-settlement serviceability threshold, a pile-cap-size-to-pile-spacing ratio of 0.5 and a pile-cap area replacement ratio of 25% are obtained for the working conditions examined in this study. These values are only applicable to the site and parameter ranges investigated herein. Pile length should be designed primarily to satisfy bearing-capacity requirements and to penetrate through soft-soil layers.

## Figures and Tables

**Table 1:** Summary of field-test scheme.

Working Condition Number	Cement-Soil Cushion Thickness (mm)	Position and Number of Reinforcement Layer
1	500	Cushion bottom One layer
2	400	Cushion bottom One layer
3	300	Cushion bottom One layer
4	300	Cushion top + bottom Two layers
5	/	300 mm reinforced gravel cushion (control group)

**Table 2:** Factors considered in numerical calculation.

Serial Number	Factor	Working Condition
1	Cement content (%) (reaction strength parameter)	4, 6, 8
2	Cushion thickness (mm)	200, 300, 500, 600
3	Number of reinforcement layers	1 layer, 2 layers, no
4	Position of reinforcement layer	Top, bottom, top + bottom, none
5	Dimension of pile cap (cm)	100 × 30, 120 × 30, 140 × 30, 160 × 30
6	Length of the piles (m)	6, 10, 15
7	Pile spacing (m)	2.0, 2.4, 2.8, 3.2

**Table 3:** Summary of numerical calculation conditions.

Serial Number	Working Condition	Cement Dosage (%)	Cushion Thickness (mm)	Number of Reinforcement Layers	Position of Reinforcement Layer	Dimension of Pile Cap (cm)	Length of the Piles (m)	Pile Spacing (m)
1	SNCL1	4	300	1	Bottom	140 × 30	10	2.4
2	SNCL2	6	300	1	Bottom	140 × 30	10	2.4
3	SNCL3	8	300	1	Bottom	140 × 30	10	2.4
4	DCHD1	4	400	1	Bottom	140 × 30	10	2.4
5	DCHD2	4	500	1	Bottom	140 × 30	10	2.4
6	DCHD3	4	600	1	Bottom	140 × 30	10	2.4
7	JJ1	4	300	0	None	140 × 30	10	2.4
8	JJ2	4	300	1	Top	140 × 30	10	2.4
9	JJ3	4	300	2	Top + bottom	140 × 30	10	2.4
10	ZMCC1	4	300	1	Bottom	100 × 30	10	2.4
11	ZMCC2	4	300	1	Bottom	120 × 30	10	2.4
12	ZMCC3	4	300	1	Bottom	160 × 30	10	2.4
13	ZC1	4	300	1	Bottom	140 × 30	6	2.4
14	ZC2	4	300	1	Bottom	140 × 30	15	2.4
15	ZJU1	4	300	1	Bottom	140 × 30	10	2.0
16	ZJU2	4	300	1	Bottom	140 × 30	10	2.8
17	ZJU3	4	300	1	Bottom	140 × 30	10	3.2

**Table 4:** Properties of the adopted geogrid.

Parameter	Transverse Direction	Longitudinal Direction
Rib width (mm)	5	3.5
Rib thickness (mm)	1.5	4.5
Ultimate tensile strength (kN/m)	≥50	≥50
Elongation at nominal tensile strength (%)	≤13	≤15
Tensile strength at 2% strain (kN/m)	≥17.5	≥17.5
Tensile strength at 5% strain (kN/m)	≥35	≥35
Aperture size (mm × mm)	33 × 33	33 × 33

**Table 5:** Mohr–Coulomb parameters of cement-treated soil cushion under different cement dosages.

Cement Dosage (%)	Cohesion c (kPa)	Friction Angle φ (°)	Dilation Angle ψ (°)	Elastic Modulus E (MPa)	Poisson’s Ratio	Unit Weight (kN/m^3^)
4	250	24	12	200	0.22	19.5
6	275	25	12.5	230	0.22	19.5
8	288	26	13	260	0.22	19.5

**Table 6:** Prestressed concrete pipe-pile size and calculation parameters.

Length of the Pile (m)	Diameter of Pile (m)	Wall Thickness (mm)	Elastic Modulus (GPa)	Pile Strength (MPa)
10	0.4	60	36	60

**Table 7:** Soil layer thickness and geotechnical parameters of foundation soils.

Foundation Soil	Thickness (m)	Compression Modulus (MPa)	Poisson Ratio	Gravity (kN/m^3^)	Cohesion c (kPa)	Friction Angle φ (°)	Dilation Angle ψ (°)
Silty clay	10	13.7	0.3	18	34.5	10.8	5
Silty sand	17.6	12.2	0.3	19	9.8	28.9	14
Silty soil	22.4	11.3	0.3	18	40	9.5	4.5
Embankment filling	6	15	0.3	20	5	30	15

**Table 8:** Model soil-thickness and calculation parameters.

Soil Layer No.	Soil Type	Thickness (cm)	Young’s Modulus (MPa)	Poisson’s Ratio	Unit Weight (kN/m^3^)
1	Soil 1	1000	16	0.3	19
2	Soil 2				
3	Soil 3				
4	Soil 4				
5	Soil 5	2240	12	0.3	19
6	Soil 6				
7	Soil 7				
8	Soil 8				
9	Soil 9	1760	23.16	0.3	19.9

**Table 9:** Measured and simulated values at representative filling heights (Case 1).

Monitoring Variable	Filling Height (m)	Measured	Simulated	Relative Error (%)
Pile-top settlement (mm)	2	11.8	12.0	1.7
Pile-top settlement (mm)	4	18.7	19.5	4.3
Pile-top settlement (mm)	6	26.8	26.9	0.4
Inter-pile soil settlement (mm)	2	14.5	17.8	22.8
Inter-pile soil settlement (mm)	4	27.9	33.7	20.8
Inter-pile soil settlement (mm)	6	43.2	44.6	3.2
Pile-top earth pressure (kPa)	2	84.7	82.4	2.7
Pile-top earth pressure (kPa)	4	231.3	216.1	6.6
Pile-top earth pressure (kPa)	6	350.5	339.8	3.1
Inter-pile soil earth pressure (kPa)	2	25.9	24.1	6.9
Inter-pile soil earth pressure (kPa)	4	38.8	42.2	8.8
Inter-pile soil earth pressure (kPa)	6	52.7	56.9	8.0

Note: These statistical metrics (R^2^, RMSE, MAPE and maximum relative error) are calculated for Case 1 only. R^2^ = 0.93; RMSE = 2.8 mm (settlement), RMSE = 10.7 kPa (earth pressure); MAPE = 8.9%; maximum relative error = 22.8%.

## Data Availability

The original contributions presented in this study are included in the article. Further inquiries can be directed to the corresponding authors.
